# Supplementary material for: Pyruvate Dehydrogenase Kinase 1 inhibition mediated oxidative phosphorylation enhancement in cartilage promotes osteoarthritis progression
Source: BMC Musculoskelet Disord. 2023 Jul 20;24:597. doi: 10.1186/s12891-023-06585-6 (PMC10357736; doi:10.1186/s12891-023-06585-6)
Supplement: Supplementary file 1 — Additional file 1. [file 12891_2023_6585_MOESM1_ESM.zip › BP.Gsea.1653623667859/GOBP_VIRION_ASSEMBLY.html]

Details for gene set GOBP\_VIRION\_ASSEMBLY[GSEA]

|  || Dataset | 55457\_collapsed\_to\_symbols.cls.cls #OA\_versus\_normal.cls.cls #OA\_versus\_normal\_repos |
| Phenotype | cls.cls#OA\_versus\_normal\_repos |
| Upregulated in class | OA |
| GeneSet | GOBP\_VIRION\_ASSEMBLY |
| Enrichment Score (ES) | 0.49600026 |
| Normalized Enrichment Score (NES) | 1.7253859 |
| Nominal p-value | 0.0 |
| FDR q-value | 0.13144803 |
| FWER p-Value | 0.999 |
Table: GSEA Results Summary

  

Fig 1: Enrichment plot: GOBP\_VIRION\_ASSEMBLY      
 Profile of the Running ES Score & Positions of GeneSet Members on the Rank Ordered List

  

| SYMBOL | TITLE | RANK IN GENE LIST | RANK METRIC SCORE | RUNNING ES | CORE ENRICHMENT || 1 | CHMP2B | charged multivesicular body protein 2B [Source:HGNC Symbol;Acc:HGNC:24537] | 129 | 0.876 | 0.0773 | Yes |
| 2 | TSG101 | tumor susceptibility 101 [Source:HGNC Symbol;Acc:HGNC:15971] | 284 | 0.763 | 0.1414 | Yes |
| 3 | SPCS1 | signal peptidase complex subunit 1 [Source:HGNC Symbol;Acc:HGNC:23401] | 303 | 0.747 | 0.2144 | Yes |
| 4 | CHMP4A | charged multivesicular body protein 4A [Source:HGNC Symbol;Acc:HGNC:20274] | 526 | 0.649 | 0.2619 | Yes |
| 5 | CHMP5 | charged multivesicular body protein 5 [Source:HGNC Symbol;Acc:HGNC:26942] | 556 | 0.638 | 0.3232 | Yes |
| 6 | CHMP2A | charged multivesicular body protein 2A [Source:HGNC Symbol;Acc:HGNC:30216] | 1269 | 0.464 | 0.3141 | Yes |
| 7 | VPS28 | VPS28 subunit of ESCRT-I [Source:HGNC Symbol;Acc:HGNC:18178] | 1522 | 0.426 | 0.3370 | Yes |
| 8 | DDX6 | DEAD-box helicase 6 [Source:HGNC Symbol;Acc:HGNC:2747] | 1687 | 0.401 | 0.3642 | Yes |
| 9 | USP6NL | USP6 N-terminal like [Source:HGNC Symbol;Acc:HGNC:16858] | 1999 | 0.361 | 0.3760 | Yes |
| 10 | CHMP6 | charged multivesicular body protein 6 [Source:HGNC Symbol;Acc:HGNC:25675] | 2140 | 0.345 | 0.3996 | Yes |
| 11 | RAB1B | "RAB1B, member RAS oncogene family [Source:HGNC Symbol;Acc:HGNC:18370]" | 2453 | 0.308 | 0.4060 | Yes |
| 12 | PC | pyruvate carboxylase [Source:HGNC Symbol;Acc:HGNC:8636] | 2629 | 0.287 | 0.4211 | Yes |
| 13 | CHMP3 | charged multivesicular body protein 3 [Source:HGNC Symbol;Acc:HGNC:29865] | 2733 | 0.277 | 0.4407 | Yes |
| 14 | APOE | apolipoprotein E [Source:HGNC Symbol;Acc:HGNC:613] | 2776 | 0.272 | 0.4646 | Yes |
| 15 | VPS4B | vacuolar protein sorting 4 homolog B [Source:HGNC Symbol;Acc:HGNC:10895] | 2783 | 0.271 | 0.4911 | Yes |
| 16 | SNF8 | SNF8 subunit of ESCRT-II [Source:HGNC Symbol;Acc:HGNC:17028] | 3037 | 0.246 | 0.4960 | Yes |
| 17 | PDCD6IP | programmed cell death 6 interacting protein [Source:HGNC Symbol;Acc:HGNC:8766] | 4956 | 0.076 | 0.3546 | No |
| 18 | VPS37C | VPS37C subunit of ESCRT-I [Source:HGNC Symbol;Acc:HGNC:26097] | 5060 | 0.068 | 0.3534 | No |
| 19 | CHMP1B | charged multivesicular body protein 1B [Source:HGNC Symbol;Acc:HGNC:24287] | 7339 | -0.105 | 0.1869 | No |
| 20 | NEDD4 | NEDD4 E3 ubiquitin protein ligase [Source:HGNC Symbol;Acc:HGNC:7727] | 7407 | -0.111 | 0.1928 | No |
| 21 | RAB1A | "RAB1A, member RAS oncogene family [Source:HGNC Symbol;Acc:HGNC:9758]" | 9604 | -0.284 | 0.0505 | No |
| 22 | CHMP7 | charged multivesicular body protein 7 [Source:HGNC Symbol;Acc:HGNC:28439] | 9723 | -0.294 | 0.0707 | No |
| 23 | MVB12B | multivesicular body subunit 12B [Source:HGNC Symbol;Acc:HGNC:23368] | 9842 | -0.305 | 0.0919 | No |
| 24 | VPS4A | vacuolar protein sorting 4 homolog A [Source:HGNC Symbol;Acc:HGNC:13488] | 10768 | -0.401 | 0.0601 | No |
| 25 | CHMP1A | charged multivesicular body protein 1A [Source:HGNC Symbol;Acc:HGNC:8740] | 11401 | -0.487 | 0.0595 | No |
| 26 | VPS37B | VPS37B subunit of ESCRT-I [Source:HGNC Symbol;Acc:HGNC:25754] | 11894 | -0.570 | 0.0781 | No |
Table: GSEA details [plain text format]

  

Fig 2: GOBP\_VIRION\_ASSEMBLY      
 Blue-Pink O' Gram in the Space of the Analyzed GeneSet

  

Fig 3: GOBP\_VIRION\_ASSEMBLY: Random ES distribution      
 Gene set null distribution of ES for **GOBP\_VIRION\_ASSEMBLY**
